# Vesicle-Like Biomechanics Governs Important Aspects of Nuclear Geometry in Fission Yeast

**DOI:** 10.1371/journal.pone.0000948

**Published:** 2007-09-26

**Authors:** Gerald Lim H. W., Greg Huber, Yoshihiro Torii, Aiko Hirata, Jonathan Miller, Shelley Sazer

**Affiliations:** 1 Department of Biochemistry and Molecular Biology, Baylor College of Medicine, Houston, Texas, United States of America; 2 Center for Cell Analysis and Modeling, University of Connecticut Health Center, Farmington, Connecticut, United States of America; 3 Department of Mathematics, University of Connecticut, Storrs, Connecticut, United States of America; 4 Department of Integrated Biosciences, Graduate School of Frontier Sciences, University of Tokyo, Kashiwanoha, Kashiwa, Chiba, Japan; University of Arizona, United States of America

## Abstract

It has long been known that during the closed mitosis of many unicellular eukaryotes, including the fission yeast (*Schizosaccharomyces pombe*), the nuclear envelope remains intact while the nucleus undergoes a remarkable sequence of shape transformations driven by elongation of an intranuclear mitotic spindle whose ends are capped by spindle pole bodies embedded in the nuclear envelope. However, the mechanical basis of these normal cell cycle transformations, and abnormal nuclear shapes caused by intranuclear elongation of microtubules lacking spindle pole bodies, remain unknown. Although there are models describing the shapes of lipid vesicles deformed by elongation of microtubule bundles, there are no models describing normal or abnormal shape changes in the nucleus. We describe here a novel biophysical model of interphase nuclear geometry in fission yeast that accounts for critical aspects of the mechanics of the fission yeast nucleus, including the biophysical properties of lipid bilayers, forces exerted on the nuclear envelope by elongating microtubules, and access to a lipid reservoir, essential for the large increase in nuclear surface area during the cell cycle. We present experimental confirmation of the novel and non-trivial geometries predicted by our model, which has no free parameters. We also use the model to provide insight into the mechanical basis of previously described defects in nuclear division, including abnormal nuclear shapes and loss of nuclear envelope integrity. The model predicts that (i) despite differences in structure and composition, fission yeast nuclei and vesicles with fluid lipid bilayers have common mechanical properties; (ii) the *S. pombe* nucleus is not lined with any structure with shear resistance, comparable to the nuclear lamina of higher eukaryotes. We validate the model and its predictions by analyzing wild type cells in which *ned1* gene overexpression causes elongation of an intranuclear microtubule bundle that deforms the nucleus of interphase cells.

## Introduction

The distinguishing characteristic of eukaryotic cells is a membrane-bound nucleus containing the genetic material. The inner and outer lipid bilayers of the nuclear envelope (NE) fuse at the nuclear pore complexes (NPCs) to form a quasi-double bilayer perforated by aqueous channels. The NE membranes and NE lumen are continuous with those of the endoplasmic reticulum (ER), making the nucleus a specialized region of the ER network [reviewed in 1], [2], [3]. Two critical differences between higher eukaryotes, such as animals, and many lower eukaryotes, including the fission yeast *Schizosaccharomyces pombe*, are: (i) Yeast lack a lamin-based structure that provides structural support to the NE of higher eukaryotes (there are no lamin orthologs in the fully sequenced genomes of fission or budding yeast [4]), but it is not known if yeast possess a shear-resistant structure functionally analogous to the eukaryotic lamina; and (ii) The NE of higher eukaryotes breaks down at mitosis, whereas in many unicellular eukaryotes, including fission yeast, it remains intact [reviewed in 5].

**Figure 1:**
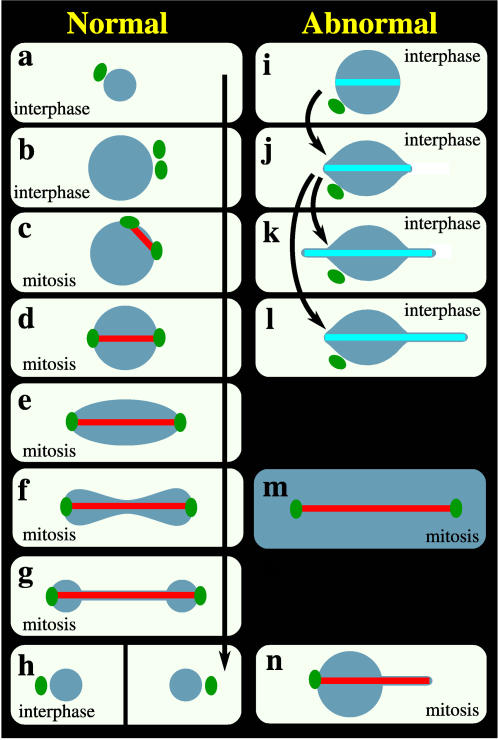
Normal nuclear geometric transformations in the life cycle of fission yeast and a catalogue of fission yeast nuclear abnormalities. In interphase (a to b), which lasts for 3–4 hr, the volume of the nucleus increases with time to twice the initial volume of ∼4π/3×1.13 µm^3^. When mitosis begins, the duplicated SPBs (b) are embedded in the NE (c) and as they assume positions on opposite sides of the nucleus (c, d) nucleate the assembly of MTs that form the mitotic spindle. As the spindle elongates (d–f) to a final length of 12–15 µm [15], the initially spherical nucleus (d), measuring ∼1.1×2^1/3^ µm in radius, is deformed consecutively into an oval (e), peanut (f), and dumbbell (g) shape before resolving into two spherical daughter nuclei (h) ∼1.1 µm in radius. Cytokinesis (h) then physically separates the nuclei into two individual cells (a) that initiate another round of cell division. (i to l) Formation and elongation of a n-MTB in interphase cells, leading to formation of one or two tethers, upon *ned1* overexpression [6]. (m) NE fragmentation during mitosis, in strains in which the Ran GTPase system is perturbed [reviewed in 35]. (n) During abnormal mitosis in the cut11-2 temperature sensitive mutant [11], the msd1 null mutant [12] or upon *mia1* overexpression [13] or laser microsurgery [14], one end of the mitotic spindle has no SPB or is not properly anchored to the SPB and, upon spindle elongation, this end induces a single tether whereas the NE appears undeformed at the other end. Dark blue: nucleus; green: SPB; red: spindle in mitotic cells; turquoise: n-MTB in interphase cells.

Early in the closed mitosis of fission yeast, the chromosomes condense but the nucleus remains quasi-spherical. The duplicated spindle pole bodies (SPBs), that nucleate formation of the mitotic spindle, become embedded in the NE. Elongation of the intranuclear mitotic spindle with spindle pole bodies (SPBs) at its ends then drives the nucleus into oblong, peanut and then dumbbell shapes (Fig. 1, c–g).

During interphase (the time between mitoses) the fission yeast nucleus increases in volume and area with no change to its quasi-spherical shape (Fig. 1, a–b). Interphase fission yeast and animal cells do not have a spindle, which typifies cells in mitosis (Fig. 1, c–g), but they have a cytoplasmic array of microtubules that are absent from mitotic cells. However, an unusual situation has been observed in the interphase nuclei of wild type cells in which overexpression of the *ned1* gene leads to formation of a nuclear microtubule bundle (n-MTB) (Fig. 1i). The n-MTB is similar to a mitotic spindle, in that it can elongate within the nucleus (Fig. 1, j–l), but it lacks SPBs at its ends [6] (compare Fig 1, c–g to i–l). It is clear that these cells are in interphase because they have cytoplasmic microtubules and unduplicated SPBs. Elongation of the n-MTB changes interphase nuclear shape from quasi-spherical to lemon, and then to lemon with one or two thin protrusions (tethers) (Fig. 1, i–l), transformations strikingly different from those seen during mitosis (Fig. 1, c–g).

We are formulating the first biomechanical model of the fission yeast nucleus, in order to investigate the properties of the NE, to provide a mechanical framework within which to understand genetic defects leading to abnormal nuclear shapes and sizes, and to test the possible influence of factors such as a shear-resistant nuclear scaffold, chromatin, SPBs, NPCs and/or the nucleolus, on nuclear division.

The starting points for the model are the mechanical properties of lipid bilayers, the well-characterized nuclear shape transformations induced by microtubule (MT) elongation during normal mitosis (Fig. 1, a–h) and abnormal (Fig. 1, m, n) mitosis in fission yeast, and the observation that MT elongation within closed lipid-bilayer vesicles [7]–[9] results in shape transformations reminiscent of those induced *in vivo* by n-MTB elongation in interphase nuclei (Fig. 1, i–l).

The model cannot yet describe the complex morphological changes of mitotic nuclei, and we discuss possible reasons for this. But, in its current form, it can describe both normal and abnormal interphase nuclear geometry, specifically the nuclear tethers induced by elongation of an n-MTB lacking SPBs at either end upon overexpression of the *ned1* gene [6]. The model also provides a novel biomechanical explanation for abnormal mitotic nuclear geometries caused by mis-regulation of the Ran GTPase [10] that results in NE breaking, by mutation or overexpression of genes that influence MT function [6], [11]–[13], or upon laser-induced MT breakage [14]. In these cases, the MT end lacking a SPB causes tether formation upon elongation but the opposite SPB-containing end shows normal nuclear curvature.

## Results

### Physical properties of the nuclear envelope that form the basis for a mechanical model of S. pombe nuclear geometry

To quantify cell cycle changes in cell volume, we compared the mean nuclear diameter of cells with a single nucleus before mitosis (*d*_i_) (Fig. 1, b–c) and in cells with two nuclei after (*d*_f_) mitosis (Fig 1g) (Table S1). We found that the mean of *d*_i_/*d*_f_ is 1.27, which is in close agreement with the expected ratio of 2^1/3^ = 1.26 for an ideal sphere that doubles in volume during interphase (Fig. 1, a to b) and then divides into two smaller ideal spheres at constant volume during mitosis (Fig. 1, c–g).

Both the nuclear volume and the NE area double in each cell cycle, but all of the volume increase occurs during interphase (Figs. 1a–1b). In contrast, the NE area doubles in two stages: a 59% increase during interphase (Figs. 1a–1b) and a further 26% increase during mitosis (Figs. 1c–1h), as dictated by simple geometry. These increases require the NE to be coupled to an external area reservoir, a characteristic that distinguishes the fission yeast nucleus from vesicles with undilated bilayers.

### Formulation of a mechanical model of S. pombe nuclear geometry

**Figure 2:**
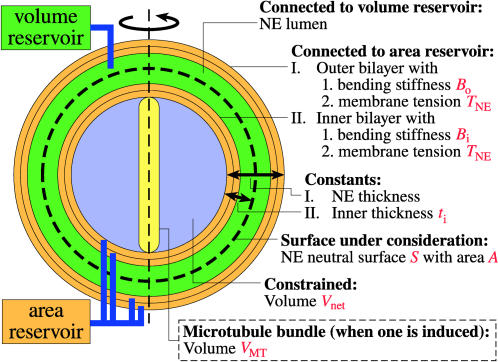
Minimal model describing the mechanics governing the geometries of interphase fission yeast nuclei and abnormal nuclei in which a n-MTB is induced.

The goal of developing a mathematical model describing the fission yeast nucleus was to determine the physical properties of the nucleus that govern its size and shape and then describe in mathematical terms their relative contributions to minimization of the free energy of the system. Our basic assumptions were that the observed geometric transformations of fission yeast nuclei occur under quasi-static conditions and that the observed geometries minimize the free energy for the NE and satisfy all relevant biophysical constraints. We formulated a free energy for the NE (described in Methods; Fig. 2), based on current understanding of fission yeast nuclear architecture [6], [11], [15]–[17] and Canham-Helfrich bilayer mechanics [1], [18]–[22], which contributed a membrane tension term and a bending resistance term, respectively (Table 1, Eq. [1]).

**Table 1:** Equations (A) and definitions of mathematical symbols (B).

	**A. EQUATIONS**
[1]	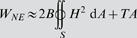
[2]	*W = W*_NE_−*PV*_net_
[3]	*V* = V_net_+V_MT_+A*t*_i_
[4]	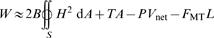
[5]	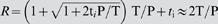
[6]	ρ≥ρ_min_ = r+*t*_i_
[7]	*T/B*≈1/(2ρ^2^) for ρ>ρ_min_
[8]	*T/B*≥1/(2ρ^2^) for ρ = ρ_min_
[9]	*T*_NE_ = T/2
	**B. DEFINITIONS**
*A*	area of S
*B*	effective NE bending stiffness
*F*_MT_	axial force of n-MTB
*H*	mean curvature of S
*L*	n-MTB or tether length
*P*	Lagrange multiplier
*r*	n-MTB radius
*R*	nuclear surface radius
ρ	tether radius
*S*	neutral surface of the NE
*T*	apparent NE tension
*T*_NE_	actual NE tension
*t*_i_	constant inner NE thickness
*V*_MT_	n-MTB volume
*V*_net_	soluble nuclear volume

#### NE membrane tension and nuclear volume constraint determine interphase nuclear geometries

The nuclear volume, (Table S1) is accounted for mathematically by imposing a volume constraint through the method of Lagrange multipliers, in which the product of the constrained volume (*V*_net_, the volume enclosed by the inner NE bilayer minus the volume of impermeable structures, such as the n-MTB (Fig. 2)) and a Lagrange multiplier (a mathematical term that constrains *V*_net_ to experimentally observed values) is subtracted from the NE free energy to form a functional to be minimized (Table 1, Eq. [2]), with the Lagrange multiplier adjusted to keep the constrained volume at its given value. In most contexts the Lagrange multiplier has no physical meaning, but, in this case, it may represent the pressure differential across the thickness of the inner bilayer.

**Figure 3:**
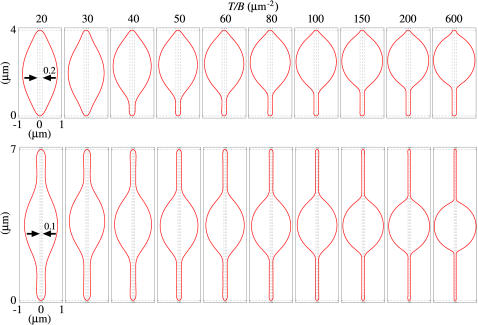
Stable (top row) and locally stable (bottom row) axisymmetric surfaces given by numerical minimization of W. The top row shows a one-parameter scan through a range of fixed *T/B* values, with the constraints *t*_i_ = 0, *V*_net_ = 4π/3 = 4.19 µm^3^, *L* = 4 µm and *r* = 0.1 µm. The values of *T/B* are, from left to right, 20, 30, 40, 50, 60, 80, 100, 150, 200, and 600 µm^−2^. These *T/B* values are within the expected physical range, since they are at least an order of magnitude smaller than that required for membrane rupture, assuming that *B* ∼4×10^−19^ J [26] and the membrane ruptures at *T* ∼2 mN/m [20]. The bottom row shows a one-parameter scan through the same range of *T/B* values as above, with the constraints *t*_i_ = 0, *V*_net_ = 4π/3 = 4.19 µm^3^, *L* = 7 µm, and *r* = 0.05 µm. Top row, left to right: *A* = 14.44, 14.06, 14.07, 13.96, 13.88, 13.81, 13.79,13.76, 13.75, and 13.74 µm^2^; *P/B* = 33.15, 51.80, 70.47, 89.28, 108.15, 146.07, 183.98, 278.71, 373.49, and 1131.32 µm^−3^. Bottom row, left to right: *A* = 17.33, 16.43, 16.04, 15.70, 15.45, 15.09, 14.84, 14.46, 14.23, and 14.11 µm^2^; *P/B* = 34.37, 53.10, 71.64, 90.51, 109.48, 147.62, 185.96, 282.39, 379.30, and 1158.77 µm^−3^. The two-tether surfaces are calculated without imposing mirror symmetry about the plane bisecting the n-MTB.

When a n-MTB is induced to form in an interphase nucleus, the total volume enclosed by the NE surface (Table 1, Eq. [3]) is the sum of the net soluble nuclear volume (*V*_NET_), the n-MTB volume and the volume of the inner NE (defined in Fig. 3). The inner thickness of the NE makes a small but finite contribution (∼5% or less) to the volume. In contrast to closed single-bilayer vesicles, which generally have different shapes, minimization of the NE free energy with a volume constraint yields stable surfaces with unique spherical shapes because a sphere surface has the lowest bending energy (8π*B*) and smallest area for a given volume.

The geometry of the nuclear surface is effectively determined by one quantity, the ratio of the apparent NE tension to a “pressure” (the Lagrange multiplier enforcing the volume constraint), since the surface's radius satisfies a Young-Laplace-type relation (Table 1, Eq. [5]). Likewise, the relationship between the NE tension (T) and the Lagrange multiplier (P) can be estimated from the experimentally determined nuclear radius.

The forces and constraints represented by Eq. [2] therefore constitute a minimal model for the nuclear volume increase at constant spherical shape during interphase of the fission yeast cell cycle (Fig. 1, a–b): this behavior is interpreted as an increase of *T*/*P* over time.

#### The fission yeast nucleus and a lipid vesicle have common and unique physical properties

During interphase, the changes in nuclear shape upon n-MTB elongation (Fig. 1, i–l) are strikingly different from normal mitotic shape changes (Fig. 1, a–h), but closely resemble those of a lipid vesicle in response to MT elongation [7]–[9].

The functional governing closed Canham-Helfrich bilayer vesicles, has the same form as that we propose here for the fission yeast nucleus, Eq. [2] [18], [19], [21], [22] but with some crucial differences: the area of a closed vesicle is fixed [21] and the analog of membrane tension for the closed vesicle is now a Lagrange multiplier without direct physical interpretation. Since the fixed area generally exceeds the minimum required to hold a given volume, closed vesicles generally adopt non-spherical, non-unique shapes [21]. In contrast, according to Eq. [2], the interphase fission yeast nucleus is physically equivalent to a vesicle with a constrained volume, with a membrane that prefers to be flat, which is coupled to an area reservoir that maintains it at a certain tension. The result is a unique, spherical nuclear shape.

#### Tether formation induced by n-MTB elongation in an interphase nucleus allows estimation of NE membrane tension

The NE cannot penetrate the n-MTB, a restriction we account for by computationally excluding the calculated surface from the volume occupied by the n-MTB. We approximate the n-MTB as an impermeable, cylindrical rod with length, radius, and hemispherical ends. In the idealized limit in which the radius approaches zero (*r*→0), the n-MTB excluded-volume constraint in Eq. [2] is replaced by the term -F_MT_L which represents the work done by the n-MTB (the magnitude of the axial force exerted at two points on the NE (F_MT_) by a n-MTB of given length (L)). This yields the final form of the equation (Table 1, Eq. [4]) in which the free energy is defined by NE membrane tension and bending resistance, MT pushing force and nuclear pressure.

When n-MTB elongation induces tether formation from the interphase NE (Fig. 1, k–l), the tether radius ρ is restricted by excluded-volume effects (Table 1, Eq. [6]). Applying results based on Canham-Helfrich lipid bilayers [23]–[25], we derive equations that relate the relationship between apparent NE tension and the effective NE bending stiffness to the tether radius (Table 1, Eqs. [7] and [8]). These equations allow us to estimate the apparent NE tension (*T*), and hence the actual bilayer membrane tension (*T*_NE_) (Table 1, Eq. [9]) since the effective NE bending stiffness can be estimated from known bilayer membrane bending stiffnesses [26]. Moreover, all of the geometrical quantities determining nuclear geometry can be measured experimentally, leaving no free parameters in the model (see Methods). In general, this means that we can use Eqs. [7] and [8] to estimate the apparent tension of the NE for all cases where tethers form from a spherical nucleus (Fig. 1, g, k, l, n).

### Applications of the model to the fission yeast nucleus

#### The model predicts that nuclear surface shape is determined by competition between NE bending resistance and membrane tension

In the hypothetical limit where the NE has no bending resistance (*B* = 0), minimization of the NE free energy is equivalent to minimization of the NE area at fixed volume and fixed n-MTB geometry. There is a unique value of the minimum energy, but it corresponds to an infinite number of stable surfaces (degenerate ground states). These surfaces are bulk spheres (bulges), with one or two tethers that tightly sheath the n-MTB. The degeneracy arises because the energy minimum does not depend on the exact position of the bulge along the n-MTB. The finite membrane tension seeks to minimize the NE area at the expense of creating three energetically unfavorable highly curved surface regions: the sphere-tether junction, the tether cap, and the cylindrical length of the tether.

*In vivo*, where the NE resists bending deformation (*B*>0) in response to MT elongation, bending elasticity competes with membrane tension by forcing the surface to adopt a shape that smoothes out these highly-curved regions at the expense of increasing the area. The relative strength of these two competing forces is determined by the ratio of the apparent NE tension (*T*) to the effective NE bending stiffness (*B*), which controls the geometry of the nuclear surface (Fig. 3). At large values of *T*/*B* (Fig. 3, *T*/*B* = 600 µm^−2^), we expect the surfaces to resemble spheres with one or two tethers when the bending stiffness (*B*) is zero. However, because in general the nuclear geometries are not known *a priori* they must be calculated using full constrained minimization. We use a novel numerical minimization technique (G. Lim H.W and G. Huber, unpublished; described in Supplemental Text S1) to obtain the predicted surfaces (Fig. 3). As expected, these surfaces became smoother and their areas increased as the NE bending resistance (*B*) became more dominant (decreasing values of *T*/*B*).

#### The model describes the relationship between the forces generated by n-MTB elongation and the resulting change in interphase nuclear geometry

We have recapitulated n-MTB elongation in an interphase nucleus by increasing its length (*L*) while keeping the other parameters fixed (Fig. S1 and extended discussion in Supplemental Text S1). We find two curves of minimum energy as functions of *L*: a one-tether global minimum, and a degenerate two-tether local minimum. The curves are each characterized by a threshold tether length, the value of which depends on the other parameters. The global-minimum curve corresponds to transformation of the initially spherical nuclear surface into a stable, one-tether surface (Fig. 1l) when *L*≥*L*_1_ (where L_1_ is the threshold n-MTB length for tether initiation). The local-minimum curve for the energy minimization is identified through appropriate selection of a starting surface with two tethers. It begins at *L* = *L*_2_ (*L*_2_>*L*_1_) (where *L*_2_ is the minimum n-MTB length at which a two-tether surface is locally stable) and corresponds to locally stable, degenerate two-tether surfaces (Fig. 1k).

When the NE bending resistance is finite, not all positions of the bulge along the n-MTB are equivalent and the ground state is no longer degenerate: A two-tether surface is locally stable and degenerate when its bulge is sufficiently far away from both ends, but transforms irreversibly into a stable, one-tether surface when the bulge is close to one end. This instability defines the threshold n-MTB length (*L*_2_). In forming one-tether surfaces, the axial force exerted by a n-MTB on the NE is highest at the tether initiation length (*L*_1_), decreases slightly during tether formation, and becomes constant with increasing tether elongation length (*L*). Since two-tether surfaces are unstable in the gap *L*_1_<*L*<*L*_2_, our model does not describe the force behavior during formation of two tethers. The force exerted by a n-MTB for locally stable two-tether surfaces (*L*≥*L*_2_) is constant and essentially equal to that for one-tether surfaces.

#### The model can be used to estimate NE membrane tension from in vivo geometry of a one-or two-tether nucleus

**Figure 4:**
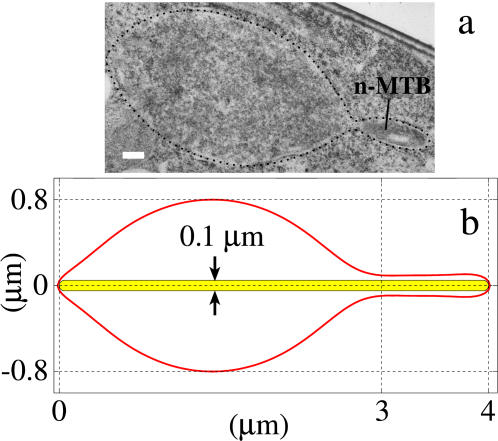
(a) Electron micrograph of thin section of an abnormally shaped nucleus (dotted outline) with a n-MTB due to overexpression of the *ned1* gene [6]. The n-MTB extends across the nucleus but is only partially visible in this section. Based on this and the other sections (not shown), we estimate that the bulge dimensions are approximately 1.6 µm×3.0 µm and the n-MTB radius is approximately 0.05 µm. Bar: 0.2 µm. (b) Predicted stable axisymmetric surface *S* with the same scale as above, obtained by adjusting *T/B* and *V*_net_ so that its bulge dimensions match the actual ones, with the constraints *t*_i_ = 0.016 µm, *L* = 4 µm, and *r* = 0.05 µm. Its size measures are *A* = 11.43 µm^2^ and *V*_net_ = 2.90 µm^3^, corresponding to *T/B* = 65 µm^−2^ and *P/B* = 134.28 µm^3^, respectively.

The bulge geometry is predicted to be effectively constant during both one-tether and two-tether elongations (Fig. S1). Therefore, it can be used as an alternative to Eqs. [7] and [8] for estimating *T*/*B*. We test this aspect of the model using the physical dimensions from electron micrographs of a typical example of a one-tether interphase nucleus with a n-MTB (one of which is shown in Fig. 4 and diagrammed in Fig. 1l), resulting from over-expression of the *ned1* gene in wild type cells [6]. Measurements from four sections of this nucleus indicate that the bulge dimensions are approximately 1.6 µm×3.0 µm and the n-MTB radius is approximately 0.05 µm (Fig 4a). We then searched for a surface *S* whose bulge dimensions matched these actual nuclear dimensions by scanning the values of *T*/*B* (apparent NE tension/effective NE bending stiffness) and *V*_net_ (soluble nuclear volume) while holding the *t*_i_ (inner NE thickness), *L* (n-MTB length), and *r* (n-MTB radius) fixed. We determined that the best match was the surface with *T/B* = 65 µm^−2^ and V_net_ = 2.90 µm^3^ (Fig. 4b). Assuming a typical value for *B* of 4.00×10^−19^ (24), the membrane tension of this NE bilayer was 0.013 mN/m, which is comparable to the tension estimated for other intracellular biological bilayers such as the Golgi and ER membranes [27].

## Discussion

We have taken a computational approach to determining the mechanical properties of the fission yeast nucleus. In order to ask how spherical nuclear shape is maintained during interphase, and to eventually understand how it is maintained during nuclear division in fission yeast (Fig. 1) we have formulated a novel biophysical model (Fig. 2; Table 1) that: i) accounts for the NE area reservoir necessary for large NE area increases during the cell cycle; ii) accounts for volumetric constraints on the nucleus; iii) describes both normal interphase nuclear geometry and n-MTB-induced tether geometries of interphase nuclei, with no free parameters; and iv) provides the basis for explaining abnormal nuclear geometries caused by mutation or overexpression of fission yeast genes involved in MT and/or SPB function.

### An area reservoir essential for NE growth is likely provided by the ER

Our model incorporates an area reservoir that is essential for doubling of the NE area during each cell cycle because lipid bilayers not connected to external reservoirs can only sustain a small area increase (≤5%) before rupturing [28]. During early anaphase B of mitosis, there is a 26% increase in NE area during the less than 5-minute transition from a single spherical nucleus to two spherical nuclei. Given the continuities of the inner and outer NE membranes and within the NE-ER network [6], [29], the ER is the most likely area reservoir, especially during mitosis, with NE area acquisition likely accomplished via membrane redistribution within this network [1]–[3], [30].

These observations are relevant to understanding the mechanical failure of the NE in the temperature sensitive pim1-d1 mutant, in which the evolutionarily conserved Ran GTPase system [reviewed in 31] is disrupted. The Ran-GTPase participates in nucleocytoplasmic transport and also has non-transport dependent roles in NE reformation after mitosis in animal cells that undergo open mitosis and in spindle assembly and the spindle assembly checkpoint in both yeast and animal cells [12], [32], [33], [reviewed in 34]. In the pim1-d1 strain, loss of NE integrity (Fig. 1m) occurs as the spindle elongates in early mitosis (Y. Torii and S. Sazer, unpublished results) and the single nucleus divides into two daughter nuclei [10], [35]. According to our model, this defect may reflect an inability to add sufficient NE area at this critical point in mitosis, which requires a 26% increase in NE area in order to prevent NE membrane tension from exceeding the rupture tension.

### Nuclear structures with shear resistance are not necessary to describe fission yeast nuclear geometries

Our model does not include shear resistance, (such as provided by the nuclear lamina of higher eukaryotes); yet nuclear shape remains essentially spherical even when the chromosomes condense at mitosis. The fact that our model can accurately describe the geometry of an interphase nucleus with a n-MTB indicates that, at least during interphase when the animal cell nuclear lamina is intact, the properties of the fission yeast NE are similar to those of a lipid bilayer: if there is a lamina analog in fission yeast, it does not influence NE geometry during interphase.

### Maintenance of nuclear volume by pressure on the NE

Another prediction of the model is that the nucleoplasm exerts pressure on the NE. The origin of this pressure is unknown and has not previously been described; however, as the nuclear pores are highly selective with respect to the proteins transported through them, osmotic pressure is a plausible source because of the high concentration of osmolytes within the nucleus. Consistent with this prediction is the observation that upon chromosome condensation in mitosis, the size and shape of the nucleus is maintained in fission yeast cells.

### Other applications and refinements of the model

There are clearly other interactions, not present in lipid vesicles, that influence nuclear division and other changes in nuclear shape such as during the horsetail phase of meiosis [36]. We are now refining the model by incorporating effects from other nuclear components, such as the chromosomes, SPBs, chromosomes directly associated with SPBs, NPCs and nucleolus. Chromosome content may influence both nuclear pressure, and the doubling of nuclear size that corresponds with the doubling of the chromosomes during each cell cycle. The NPCs, which are distributed throughout the NE and in the membrane of the tethers may directly influence the mechanical properties of the membrane and may account for the slight bulge seen at the tip of the tether by electron microscopy but not in the model (Fig. 4). Our initial focus is on the influence of the SPB because experimental evidence suggests that this structure embedded in the NE may have a direct influence on nuclear size and shape during mitosis. Elongation of MTs inside of the nucleus induces tether formation only at the end(s) lacking SPBs (compare Fig. 1, panels c–g to k, l, n): during interphase in which the unduplicated SPB is in the cytoplasm (Fig. 1, a, h), *ned1* overexpression [6] induces formation of one or two tethers (Fig. 1, k,l). During mitosis, in the cut11-2 temperature sensitive mutant [11], the msd1 null mutant [12], or upon laser-induced MT breakage [14] or *mia1* overexpression [13], spindle ends lacking a SPB or spindle microtubules not properly anchored to the SPB induce formation of a single tether, whereas the opposite end, that retains a SPB, does not deform the NE (Fig. 1, n).

## Methods

### Electron Microscopy

Thin sections of cells prepared using a previously described freeze-substitution method [37] were viewed on an electron microscope (H-7600, Hitachi Co., Tokyo, Japan) at 100 kV.

### NE free energy model

The NE area and lumen volume necessarily increase during the fission-yeast cell cycle. Thus, the NE bilayers must have an area source and the NE lumen must have a volume source. The outer NE bilayer and NE lumen are continuous, respectively, with the membrane and lumen of the ER, since the NE and ER are a single membrane system in the cell. We assume that the ER membrane is the exclusive source of new NE membrane material. It follows that the ER lumen is the NE lumen's volume reservoir.

In this scenario, the inner NE bilayer draws extra area, in the form of lipids, from the outer NE bilayer at the nuclear pores, where the two bilayers are continuous, and the outer bilayer draws extra area from the ER membrane. This is represented by the coupling of the two bilayers to an area reservoir that maintains the membrane tension of both bilayers at *T*_NE_>0. Fluid bilayers have a rupture tension in the range ∼1 to 25 mN/m [20], so *T*_NE_ has an upper bound as low as 1 mN/m. By our quasi-static assumption, the aforementioned lipid transfer and volume transfer from the ER lumen to the NE lumen are much quicker than mechanical equilibration of the nucleus.

The overall effect of any short-range interbilayer repulsion, the constraint on the local NE geometry imposed by the NPCs, the high NE area-to-NE lumen volume ratio, and the coupling of the NE lumen to a volume reservoir, is to keep the NE thickness (defined in Fig. 2) approximately uniform, at an average value of 32 nm (about six times the bilayer thickness). This allows us to assume a constant interbilayer separation.

Fluid bilayers and, thus, the NE have bending elasticity [21]. We omit the topological and submicron-bending contributions of the NE-ER junctions, NPCs, and SPBs to the NE bending energy, so that we can approximate the NE as two closed, axisymmetric bilayers with fixed interbilayer separation. We assume that the bending energy for each bilayer has the zero-spontaneous curvature Canham-Helfrich form [18], [19].

The bending resistance and membrane tension of the NE thus give rise to a NE free energy of the form *W*_NE_ = 2*B*_i_+*_S_*_i_ *H*_i_^2^ d*A*_i_+2*B*_o_+*_S_*_o_ *H*_o_^2^ dA_o_+*T*_NE_ (*A*_i_+*A*_o_), where *B*_i_ (*B*_o_), *S*_i_ (*S*_o_), *H*_i_ (*H*_o_), and *A*_i_ (*A*_o_) are the inner (outer) bilayer bending stiffness, neutral surface, mean curvature, and neutral-surface area, respectively. We choose to represent the NE by its neutral surface *S*. We choose *S* to be halfway between the two bilayer neutral surfaces and assume that the inner and outer bilayers have the same bending stiffness (*B*_i_ = *B*_o_), thus enabling us to cast in the form *W*_NE_≈2*B*+*_S_ H* ^2^ d*A*+*TA*, where *H* and *A* are the mean curvature and area of *S*, respectively, and we refer to *B*≡*B*_i_+*B*_o_ = 2*B*_i_ = 2*B*_o_ and *T*≡*T*_NE_ as the effective bending stiffness and apparent tension of the NE, respectively.

### Parameters of mechanical model

When a n-MTB forms, the expected nuclear geometry is characterized by five parameters: *L*, *r*, *t*_i_, one of (*T*/*B*, *A*), and one of (*P/B*, *V*_net_). *L*, *r*, *t*_i_, *A*, and *V*_net_ are all geometric quantities that can be determined experimentally. Consequently, there are no free parameters in our model, allowing for a stringent experimental test.

## Supporting Information

Text S1 (0.06 MB DOC)

Table S1 Comparison of pre-mitosis and post-mitosis nuclear diameter
(0.02 MB XLS)

Figure S1 Minimal free energy, axial force of the n-MTB, area of the NE neutral surface, and geometry of the NE neutral surface as a function of the n-MTB length with constraints. Minimum NE free energy WNE (I), axial force FMT of the n-MTB (II), area A of the NE neutral surface S (III), and geometry of S (a–h and g′–h′) as a function of the n-MTB length L, with the constraints T/B = 40 µm-2, ti = 0, Vnet = 4π/3 = 4.19 µm3, and r = 0.1 µm. FMT is the slope ∂WNE/∂L of the curve of minimum WNE as a function of L. The unit of length, L0 ≈ 2.01 µm, is the length at which the n-MTB begins to push on the NE. The blue and red dots denote data points given by our numerical minimization method, corresponding to formation of stable and locally stable surfaces, respectively. The solid lines for L≤L0 are described by the formulae in the Supporting Information section “Mechanical behavior during n-MTB elongation.” The solid and dashed lines for L>L0 are spline fits to the data for the stable and locally stable surfaces, respectively.
(0.33 MB TIF)
