# Supplementary material for: Vesicle-Like Biomechanics Governs Important Aspects of Nuclear Geometry in Fission Yeast
Source: PLoS One. 2007 Sep 26;2(9):e948. doi: 10.1371/journal.pone.0000948 (PMC1993828; doi:10.1371/journal.pone.0000948)
Supplement: Text S1 — (0.06 MB DOC) [file pone.0000948.s001.doc]

**Supplemental Text S1**

***Numerical Minimization Technique***

The numerical minimization method is a based on simulated annealing, and specialized to the equilibrium states of axisymmetric membrane surfaces. The surface is approximated as the surface of revolution of a one-dimensional chain of vertices and edges. The vertices and edges are treated as the infinitesimal limits of circles and their tangentially linking line segments, respectively. When considered as a surface of revolution, the resulting stack of infinitesimal tori and spheres, linked by truncated cones, allows for the formulation of a discrete form of our continuum elastic energy. The surface is deformed by allowing random displacements of vertices (subject to the boundary conditions). Re-parametrization invariance enables vertices to drift freely in the tangential direction, and this is exploited to maintain numerical accuracy. Vertex displacements are accepted or rejected according to a Metropolis algorithm, and the corresponding computational temperature (unrelated to physical temperature) is governed by a heuristic annealing schedule.

***Mechanical behavior during n-MTB elongation***

At *L* ≤ *L*_0_, the excluded-volume effect of the n-MTB gives rise to a slight, uniform inflation of surface *S* with increasing *L*. This translates into a force that can be calculated analytically: *A* = 4π(*R*_in_ + *t*_i_)^2^ and *W*_NE_ = *TA* + 8π*B*, so *F*_MT_ = *T*(∂*A*/∂*L*) = 2π*T*(*R*_in_ + *t*_i_)*r*^2^/*R*_in_^2^, where R_in_ = (3*r*^2^*L*/4 − *r*^3^/2 + *R*_net_^3^)^1/3^ and *R*_net_ is defined by *V*_net_ ≡ 4π*R*_net_^3^/3. At *L* = *L*_0_, *F*_MT_(*L*_0_) = 1 pN, assuming a reasonable value for *B* of 4 X 10^−19^ J . For *L* > *L*_0_, our numerical minimization gives data points denoted by blue and red dots in Fig. S1, corresponding to formation of one- and two-tether surfaces, respectively. The solid and dashed lines are spline fits to our one-tether and two-tether data, respectively. Clearly, the area increases with n-MTB elongation. A two-tether surface is slightly larger in area and, thus, slightly higher in energy than a one-tether surface at the same parameter values.

In the interval *L*_0_  < *L* ≤ *L*_1_, *F*_MT_ increases and the initially spherical surface undergoes mirror-symmetric deformation (Fig. S1, a to c). At *L* > *L*_1_, a tether forms (Fig. S1, c to e) and elongates (Fig. S1, e to h) from one end of the lemon-shaped bulge. The bulge geometry is essentially constant during tether elongation. *F*_MT_ decreases during tether formation, then rises slightly and becomes essentially constant during tether elongation.

Two-tether surfaces are found at *L* ≥ *L*_2_, through careful selection of the starting surface for the energy minimization. Like the one-tether case, the bulge geometry and *F*_MT_ are effectively constant in two-tether elongation (Fig. S1, g' to h'). Two-tether surfaces are not part of the sequence of geometric transformations from a sphere, since they are unstable in the interval *L*_1_ < *L* < *L*_2_ and higher in energy than their respective one-tether counterparts when *L* ≥ *L*_2_. The constant *F*_MT_ at large *L* in the two-tether case is slightly less than that in the one-tether case.

That the one-tether force is slightly larger than the two-tether force is a general occurrence for all *T*/*B* values that allow the tether radius *ρ* to exceed *ρ*_min_ (Table 1, Eq. 3) and, thus, the bulge volume to be transferred to the tether as *L* increases. Because of volume reduction in the bulge, the two-tether and one-tether surfaces eventually transform into the unique, limiting surface at large *L* that resembles a cotton bud and the slope of the two-tether energy curve must be less than the slope of the one-tether energy curve for them to merge into one at large *L*. In reality, *L* is limited by the cell wall and *V*_net_/*L* cannot be infinitesimal along the entire length of the n-MTB because of excluded volume effects of the chromosomes and nucleolus. At realistic values of *L*, the bulge volume reduction is insignificant and the bulge geometry is essentially unchanged.

If *T*/*B* is so large that the tether hugs the n-MTB tightly (Table 1, Eq. 4), there is no volume transfer from the bulge to the tether (the bulge geometry is exactly constant but slightly different under one- and two-tether elongation), the increase in *A* in both cases comes only from the linear increase in area of the tether(s) with *L* (at the same rate of ∂*A*/∂*L* = 2π*ρ*_min_), and, similarly, the increase in bending energy in both cases comes only from the linear increase in bending energy of the tether(s) with *L* (at the same rate of ∂*W*_b_/∂*L* = π*B/ρ*_min_). Consequently, the one-tether and two-tether energy curves for *L ≥ L*_2_ are parallel straight lines (identical and constant slope *F*_MT_ ≡ ∂*W*_b_/∂*L + T*(∂*A*/∂*L*) *=* π*B/ρ*_min_ + 2π*Tρ*_min_) that are slightly separated (arising from the slight difference in bulge geometry between the two cases).

**Methods**

**Quantification of nuclear volume**

Images of living cells expressing GFP-tagged nuclear reporter proteins [1] were collected using a deconvolution microscope (Applied Precision, Issaquah, Washington) and captured by a Micromax 1300 camera (Roper Scientific) using SoftWoRx software. Because the nucleus is not perfectly spherical [2] (although we refer to it as such for simplicity in the text), the minimum and maximum diameters of 12 nuclei just prior to and just after division were measured from fluorescence images of a section through the middle of each nucleus, and used to calculate the pre-mitosis mean diameter *d*_i_, post-mitosis mean diameter *d*_f_, and the ratio *d*_i_/*d*_f_.

**REFERENCES**

1. Yoshida M, Sazer S (2004) Nucleocytoplasmic transport and nuclear envelope integrity in the fission yeast *Schizosaccharomyces pombe*. Methods Cell Sci 33: 226-238.

2. Tran PT, Marsh L, Doye V, Inoue S, Chang F (2001) A mechanism for nuclear positioning in fission yeast based on microtubule pushing. J Cell Biol 153: 397-411.

## Supplemental Figures

## Figure S1.

## Minimal free energy, axial force of the n-MTB, area of the NE neutral surface, and geometry of the NE neutral surface as a function of the n-MTB length with constraints

Minimum NE free energy *W*_NE_ (I), axial force *F*_MT_ of the n-MTB (II), area *A* of the NE neutral surface *S* (III), and geometry of *S* (a–h and g'–h') as a function of the n-MTB length *L*, with the constraints *T*/*B =* 40 μm^-2^, *t*_i_ = 0, *V*_net_ = 4π/3 = 4.19 μm^3^, and *r* = 0.1 μm. *F*_MT_ is the slope ∂*W*_NE_/∂*L* of the curve of minimum *W*_NE_ as a function of *L*. The unit of length, *L*_0_ ≈ 2.01 μm, is the length at which the n-MTB begins to push on the NE. The blue and red dots denote data points given by our numerical minimization method, corresponding to formation of stable and locally stable surfaces, respectively. The solid lines for *L* ≤ *L*_0_ are described by the formulae in the Supplemental Text S1 section entitled "Mechanical behavior during n-MTB elongation". The solid and dashed lines for *L* > *L*_0_ are spline fits to the data for the stable and locally stable surfaces, respectively.

**Supplemental Tables.**

**Table S1. Comparison of pre-mitosis and post-mitosis nuclear diameter.**
