# Supplementary figures and images for: Vesicle-Like Biomechanics Governs Important Aspects of Nuclear Geometry in Fission Yeast
Source: PLoS One. 2007 Sep 26;2(9):e948. doi: 10.1371/journal.pone.0000948 (PMC1993828; doi:10.1371/journal.pone.0000948)

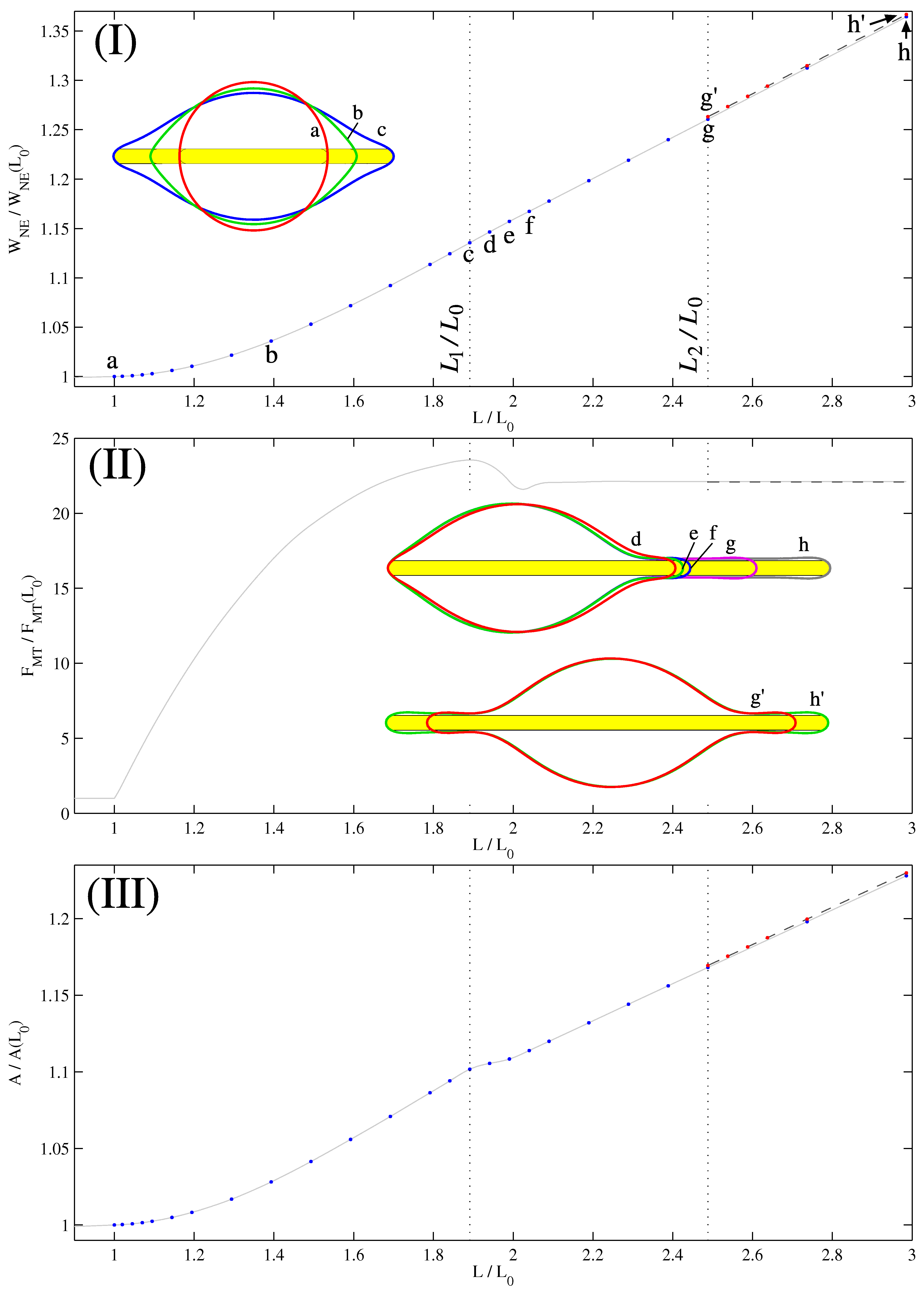

Supplement: Figure S1 — Minimal free energy, axial force of the n-MTB, area of the NE neutral surface, and geometry of the NE neutral surface as a function of the n-MTB length with constraints. Minimum NE free energy WNE (I), axial force FMT of the n-MTB (II), area A of the NE neutral surface S (III), and geometry of S (a–h and g′–h′) as a function of the n-MTB length L, with the constraints T/B = 40 µm-2, ti = 0, Vnet = 4π/3 = 4.19 µm3, and r = 0.1 µm. FMT is the slope ∂WNE/∂L of the curve of minimum WNE as a function of L. The unit of length, L0 ≈ 2.01 µm, is the length at which the n-MTB begins to push on the NE. The blue and red dots denote data points given by our numerical minimization method, corresponding to formation of stable and locally stable surfaces, respectively. The solid lines for L≤L0 are described by the formulae in the Supporting Information section “Mechanical behavior during n-MTB elongation.” The solid and dashed lines for L>L0 are spline fits to the data for the stable and locally stable surfaces, respectively. (0.33 MB TIF) [file pone.0000948.s003.tif]
